# A Zn(ii) metal–organic framework based on bimetallic paddle wheels as a luminescence indicator for carcinogenic organic pollutants: phthalate esters†

**DOI:** 10.1039/c9ra08417g

**Published:** 2019-11-13

**Authors:** Ming Ze Wu, Zhi Long Ma, Jian Yun Shi, Li Tian

**Affiliations:** Tianjin Key Laboratory of Structure and Performance for Functional Molecules, Tianjin Normal University Tianjin 300387 P. R. China lilytianli@hotmail.com; Key Laboratory of Inorganic–Organic Hybrid Functional Materials Chemistry, MOE, College of Chemistry, Tianjin Normal University Tianjin 300387 P. R. China

## Abstract

Based on the multifunctional ligand 3-(1*H*-1,2,4-triazol-1-yl)isophthalic acid (H_2_TIA), a three-dimensional coordination polymer, namely {[Zn(TIA)]·DMA}_*n*_ (Zn-1) was synthesized solvothermally. Single-crystal X-ray diffraction analyses confirmed that Zn-1 is a 3D framework composed of binuclear Zn_2_ paddle wheels with one-dimensional channels long the *a* direction. Further topological analyses revealed that MOF Zn-1 existed as a (3,6)-connected *rtl* binodal net {4·6^2^}^2^{4^2^·6^10^·8^3^}. Furthermore, the luminescence explorations indicate that complex Zn-1 is the first MOF for luminescent probing of phthalate esters (carcinogenic organic pollutants) with a high quenching-efficiency constant and low fluorescence-detection limit.

## Abstract 2

A three-dimensional coordination polymer {[Zn(TIA)]·DMA}_*n*_ (Zn-1) with *rtl* binodal topology has been synthesized. In addition, Zn-1 is the first MOF for luminescent probing of phthalate esters.

## Graphical abstract

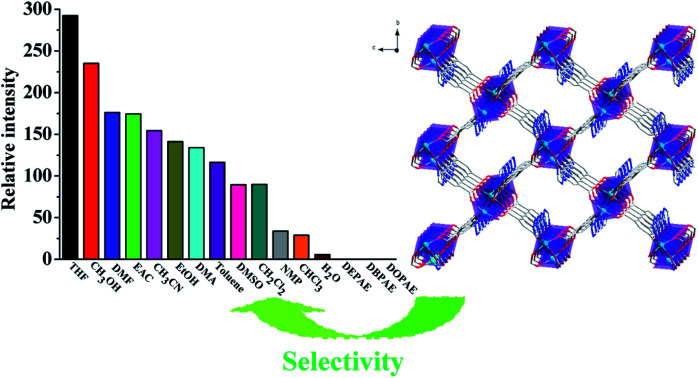

## Introduction

Owning to their appealing architectures and potential application value in catalysis,^1^ gas storage/separation,^2^ luminescence,^3^ and so on,^4^ metal–organic frameworks (MOFs), especially, those with large cavities or channels have attracted tremendous attention. In particular, a great deal of research has focused on exploring their applications in luminescence sensing, which in turn can be used to detect environmental pollutants selectively and sensitively. Distinguished from conventional carbon-based materials and porous zeolites, the ordered structure, the aperture and shape of the pore, along with the functionalities of MOFs are controllable.^5^ For example, the structure of the MOFs can be devised and regulated through reasonably selecting and adjusting of organic linkers and metal salts or clusters. Even so, the initiators characteristics and properties would heavily take impact on the final structures of coordination polymer. Besides, chemical reaction conditions, such as solvent, pH value, temperature, counter ions and so on, would also create impress influence on their final structure.^6^ Comparatively speaking, the rational selection of an appropriate organic ligand with a certain spatial configuration and active sites is considered to be the most effective strategy for constructing MOFs with the anticipated topological structures and desirable functions.

Due to the tunable channel size, large surface area and functional sites, MOFs always display amazing interaction properties between the coordination polymers and the analytes, and that makes MOFs the very sensible sensors of much more excellent properties than other luminescent materials.^7^ In particular, MOFs which have d^10^ or 4f metal centers always exhibit fascinating luminescent properties, they are considered as potential chemosensors, such as selective detectors for anions,^3*f*,8^ heavy metal cations,^9^ vapours,^10^ toxic organic molecules,^3*d*,9*d*,9*e*,11^ and biomolecules.^3*e*,12^ While, an unmet challenge is to design MOFs for quick recognizing persistent toxic organics, especially for phthalate esters (carcinogenic organic pollutants), which are believed having severe impacts on environmental protection and public health.

Phthalate esters (PAEs) are a class of organic chemical reagents, which were already and are being extensively used in commercial and industrial plastics additives. As one of the most produced and consumed chemicals in the world, PAEs undoubtedly exist in atmosphere,^13^ water,^14^ soil,^15^ sediment.^16^ And more seriously, their metabolites were reported already been found in human urine and serum.^17^ Recent researching results have shown that PAEs also may have potential carcinogenesis effects on human and animals.^18^ In addition, studies have also shown a correlation between PAE levels and precocious breast development, long-term exposure to PAE for women can lead to endometriosis and gestational duration.^19^ In addition, Gao's research group had also found that PAEs may cause central nervous system depression and obvious renal injury.^20^ Therefore, PAEs have posed great potential and obvious threaten to human and wildlife reproduction. Heavy efforts have already been taken to diminish and erase the pollution and ruin from PAEs. Precisely and rapid detection of PAEs, especially of trace concentration and amount, was believed the first and key step to success.

**Scheme 1:**
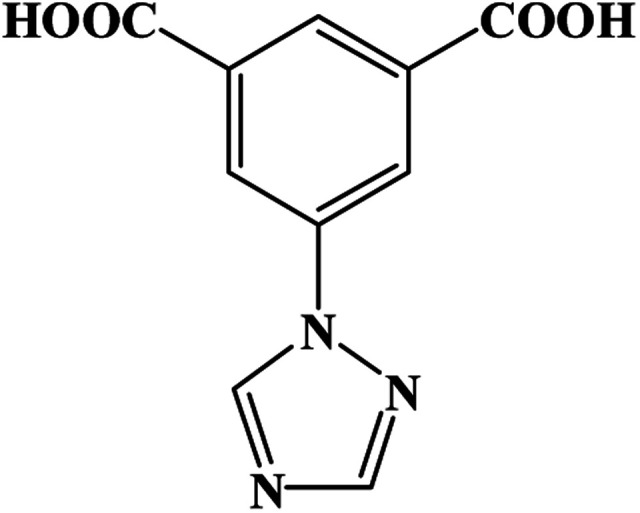
The structure of the ligand 3-(1*H*-1,2,4-triazol-1-yl)isophthalic acid (H_2_TIA).

An effective method was processed for the controllable synthesis of MOFs as phthalate esters sensor, in which, aromatic organic molecules with non-bonded functional ligand sites were selected as the precursors. The aromatic ring of the precursors has fluorescent properties, and the nonbonded functional sites will interact with d^10^ or 4f metal centers which will enhance fluorescence properties. Lately, 1,2,4-triazole and its derivatives with several N-donor atoms, as well as aromatic polyacids with several O-donor atoms are widely used to construct novel MOFs. Corresponding, a great number of coordination polymers with novel architectures, higher stabilities and peculiar functions have been reported successively.^11*a*,21^ In this context, a multi-functional organic linker 3-(1*H*-1,2,4-triazol-1-yl)isophthalic acid (H_2_TIA), which contains long π–π conjugate chain was selected as the MOFs' precursor, for it combines the advantages of the two kinds of functional groups (triazole groups and aromatic carboxylic acid groups). Besides, d^10^ metal centers such as Zn^2+^, Ag^+^, and Cd^2+^, were usually adopted in synthesizing of luminescent MOFs, because these cations can adjust the emission wavelength and intensity of organic linkers. A new MOF {[Zn(TIA)]·DMA}_*n*_ (Zn-1) with one-dimensional channels was successfully obtained from H_2_TIA and Zn(NO_3_)_2_, which showed as a 3D framework with *rtl* topology. Fluorescence recognition experiments demonstrate that micro-porous framework of Zn-1 has high efficiency in detecting phthalate esters under ambient conditions through “turn-off” luminescent detection (Scheme 1).

## Experimental details

### Materials and physical measurement

Most of the starting materials and solvents were commercially available and used without further treatment, except that the solvent dimethyl sulfoxide (DMSO) was treated by 4A molecular sieve and distilled under vacuum. 3-(1*H*-1,2,4-triazol-1-yl)isophthalic acid (H_2_TIA) was synthesized according to the literature methods.^22^ Elemental analyses for C, H, and N were taken on a PerkinElmer elemental analyzer. Infrared spectra were determined on a Bruker TENOR 27 spectrometer in the region of 4000–400 cm^−1^. Powder X-ray diffraction patterns (PXRD) were carried out on a D/Max-2500 X-ray diffractometer with Cu-Kα radiation (*λ* = 1.5406 Å) at room temperature. UV-vis spectra were collected on a UV-2600 spectrophotometer. Thermogravimetric test (TG) was measured on a NETZSCH TG 209 instrument under N_2_ atmosphere with the heating rate controlled at 10 °C min^−1^. Emission/excitation spectra were recorded on F97pro fluorescence spectrometer. Gas adsorption isotherms were performed by a volumetric method on a Micromeritics ASAP 2020HD88 surface area and pore analyzer.

### Preparation of {[Zn(TIA)]·DMA}_*n*_ (Zn-1)

H_2_TIA (17.5 mg, 0.075 mmol), Zn(NO_3_)_2_·4H_2_O (30 mg, 0.1 mmol) and 6 mL CH_3_OH–DMA (2 : 4, in v/v) were added in turn into a 15 mL Teflon-lined stainless steel vessel, and then the mixture was heated at 80 °C for three days. After that, the autoclave was cooled to room temperature at a rate of 1.2 °C h^−1^. Colorless rectangular crystals Zn-1 (20.54 mg) were obtained (yield 71% based on H_2_TIA). Elemental analysis for C_14_H_14_N_4_O_5_Zn (383.66): C 42.42, H 3.68, N 14.61%. Found: C 42.68, H 3.58, N 14.24%. IR (KBr pellet): 3503 (b), 3413 (b), 2360 (s), 1654 (s), 1623 (s), 1523 (w), 1480 (w), 1486 (w), 1374 (m), 1288 (s), 1159 (m), 1084 (m), 1055 (w), 858 (m), 720 (m), 546 (m) cm^−1^.

### Luminescence sensing experiments

The finely pressed powder samples of MOF Zn-1 (0.2 mg, optimum dosage of Zn-1 was determined by fluorescence test, Fig. S6†) were added in organic solvents (5 mL) or water. The mixture was firstly ultrasonicated for half an hour to form a stable suspension and then used for fluorescence experiment. The selected solvents are esters (ethyl acetate (EAC), diethyl phthalate ester (DEPAE), di-*n*-butyl phthalate ester (DBPAE), di-*n*-octyl phthalate ester (DOPAE)), alcohols (methanol, ethanol), amides (*N*,*N*-dimethyl formamide (DMF), *N*-methyl-2-pyrrolidone (NMP), *N*,*N*-dimethylacetamide (DMA)), methyl chloride (CH_2_Cl_2_, CHCl_3_), nitriles (CH_3_CN), dimethyl sulfoxide (DMSO), cyclic ether (tetrahydrofuran (THF)), aromatic hydrocarbon (toluene) and H_2_O. For the fluorescence quenching experiments to carcinogenic organic pollutants, phthalate esters with different volumes (μL) were added into the mixture of standard Zn-1 (0.2 mg) emulsion in DMF (5 mL).

## Results and discussion

### Crystal structure descriptions

**Fig. 1:**
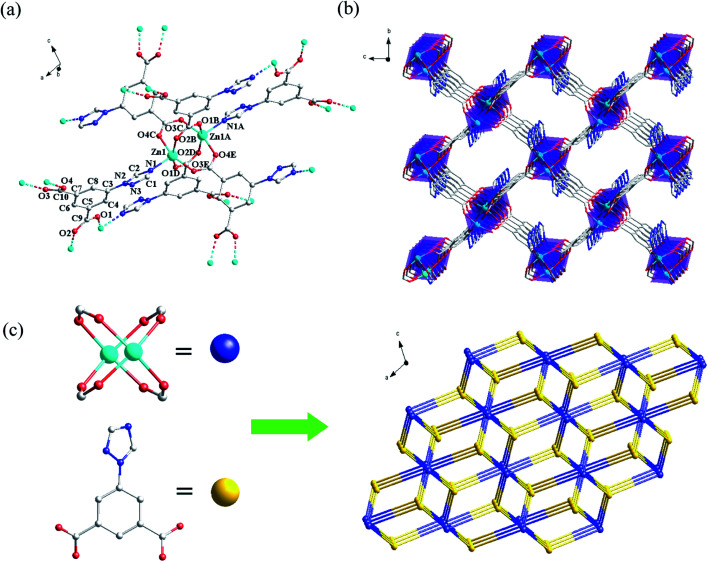
(a) The coordination environment of Zn^II^ ions in compound Zn-1. Hydrogen atoms were omitted for clarity. Symmetry operator: A = 1 − *x*, −0.5 + *y*, 1.5 − *z*; B = *x*, *y*, 1 + *z*; C = 1 + *x*, 1.5 − *y*, 0.5 + *z*; D = 2 − *x*, 1 − *y*, 1 − *z*; E = 2 − *x*, 1 − *y*, 2 − *z*. (b) The 3D framework of Zn-1 viewed along *a* direction. (c) Topology network of the framework Zn-1.

Crystal structure of {[Zn(TIA)]·DMA}_*n*_ (Zn-1). Complex Zn-1 crystallizes in the monoclinic *P*2_1_/*c* space group (Table S1, ESI†). In Zn-1, each asymmetric unit is composed by a Zn^2+^ ion, one TIA^2−^ ligand and a free DMA. As displayed in Fig. 1a, the central Zn^2+^ ion existed in a rare penta-coordinating mode finished by one N and four O donors from five different TIA^2−^ ligands to form a tetragonal pyramidal polyhedron configuration. Two Zn^2+^ ions are tetra-bridged by four μ_2_-η_1_:η_1_ carboxylate groups which come from four different TIA^2−^ ligands, to form a binuclear core with the Zn–Zn distance of 2.967(2) Å (Fig. 1a). The binuclear coordinating units are further connected by carboxylate groups and triazole N atoms of TIA^2−^ ligands to form a 3D framework (Fig. 1b), in which one-dimensional channels exist along *a* direction. In Zn-1, the triazolyl ring, the two carboxylate groups in the same TIA^2−^ ligand are not completely coplanar with the central phenyl ring.

In the 3D framework, if each Zn_2_ binuclear unit is treated as a six-connected node and each TIA^2−^ is regarded as a three-connected node, Zn-1 can be regarded as a (3,6)-connected *rtl* binodal net, with the short (Schläfli) vertex symbol of {4·6^2^}^2^{4^2^·6^10^·8^3^}, which is shown in Fig. 1c. By using the program of PLATON, the ratio of the accessible porous volume (utilizable) in every unit cell is calculated as 53.5%.

### PXRD, thermal stability and sorption properties

As shown in Fig. S2 (ESI†), powder X-ray diffraction patterns for compound Zn-1 were carried out to confirm the phase purity of crystalline materials. The main peak positions of the experiment data coincide with the corresponding simulated ones, suggesting the good phase purity of the bulk crystals Zn-1.

For the purpose of detecting the thermal stability of the framework in Zn-1, thermogravimetric analyses (TGA) was performed at the heating rated of 10 °C min^−1^ under a N_2_ atmosphere in the temperature range of 30–800 °C. The TGA curve (Fig. S4†) of Zn-1 displays as a two-step weight loss, the first step weight loss of 22.93% before 220 °C (calcd 22.68%) contributed by the loss of free DMA molecule in the lattice. The second step weight loss attributes to the collapse of the 3D framework at 366 °C, which demonstrates the framework is relatively stable.

The permanent porosity of MOF Zn-1 was further detected by the N_2_ sorption isotherm, which was carried out at 77 K. As displayed in Fig. S5,† it was a type-I isotherm with a BET surface area of 31.46 m^2^ g^−1^ (Langmuir surface area 47.83 m^2^ g^−1^).

### Photoluminescent properties of Zn-1 and H_2_TIA in solid state

Organic compounds which contain wide π–π conjugated systems and aromatic rings, along with their relevant metal–organic coordination polymers, have aroused extensive interest of chemists owning to their distinctive luminescent properties and latent applications in fluorescent devices, such as light-emitting diodes (LEDs). The architecture of novel coordination polymers by reasonable selection of conjugated organic connectors and transition metal centers (such as Zn^2+^, Cd^2+^, and Ag^+^) may be one of the most efficacious methods to obtain new kinds of luminescent materials, because of the capability to adjust the emission wavelength and intensity of organic linkers. Herein, the photo-luminescent properties of the aromatic H_2_TIA and its relevant complex Zn-1 in the solid state have been investigated.

**Fig. 2:**
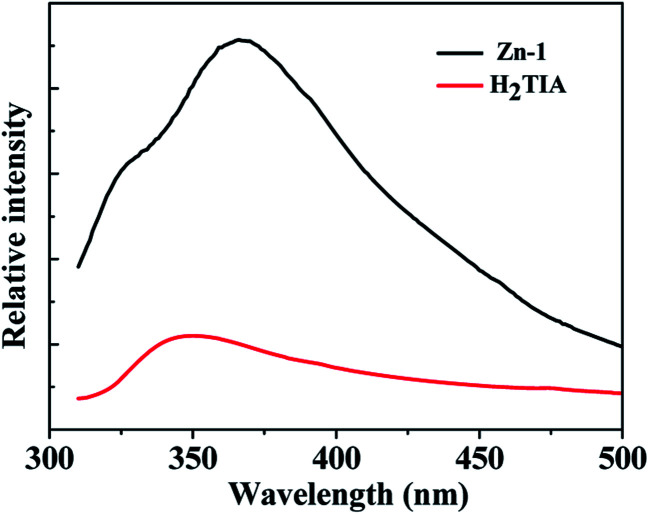
Fluorescence spectra for H_2_TIA and Zn-1 in solid-state at the excitation wavelength of 290 nm.

At ambient temperature, the organic linker H_2_TIA exhibits a broad emission band with the maximum at 350 nm (*λ*_ex_ = 290 nm), which is assigned as π → π* transitions caused by the large conjugated aromatic triazole and phenyl rings (Fig. 2).^23^ On comparison, Zn-1 shows an intense fluorescence emission band centered at 366 nm (*λ*_ex_ = 290 nm), which is contributed by the intraligand emission. Compared with organic ligand H_2_TIA, the enhancement of fluorescence intensity is attributed to that the rigidity of the ligand significantly increases by metal–ligand coordination, and the non-radiative decay between intraligand transition states decreases accordingly. For coordination polymers, the emission band based on organic ligand is preferable, since the band gaps of host material can be altered along with exchanged guest molecules diffusing into the channels based on host–guest interaction, thus resulting in different fluorescent response on the basis of the change of luminescent intensity or position.^24^ Consequently, MOF Zn-1 may be used as a fluorescent sensor owning to its structural particularities which contain 1D channels decorated by a number of open coordinating locations. In addition, MOF Zn-1 is insoluble in general organic solvents, therefore its selective sensing ability was investigated for small molecular organic solvents as well as the firmness of the framework and the durability of the micropore.

### Luminescent sensing properties of Zn-1 to phthalate esters

**Fig. 3:**
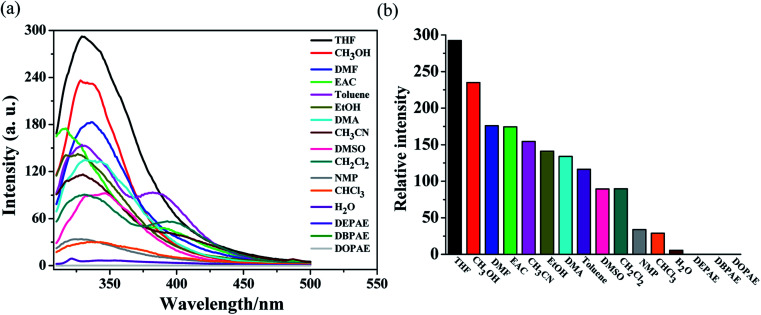
(a) Emission spectra of Zn-1 dispersed in different organic solvents when excited at 290 nm. (b) Relative luminescence intensities in various solvents at the wavelength of 325 nm.

Grounded samples of MOF Zn-1 (2 mg) were immersed in selected solvents (5 mL), which included CH_3_OH, EtOH, EAC, phthalate esters (DEPAE, DBPAE, DOPAE), DMF, DMA, CH_2_Cl_2_, CHCl_3_, CH_3_CN, DMSO, toluene, THF, and H_2_O. As exhibited in Fig. 3, the testing results showed that the fluorescence intensities of complex Zn-1 were solvent dependent. For phthalate esters (DEPAE, DBPAE, DOPAE), obvious luminescence quenching phenomena to Zn-1 were observed, with the highest quenching efficiencies as 99.96%, 99.98%, and 99.97%, respectively. However, other solvents exerted a certain degree of attenuation to the luminescence intensities of Zn-1. These results demonstrate that Zn-1 may be potential fluorescence sensors for phthalate esters (PAEs, a class of carcinogenic organic pollutants).

**Fig. 4:**
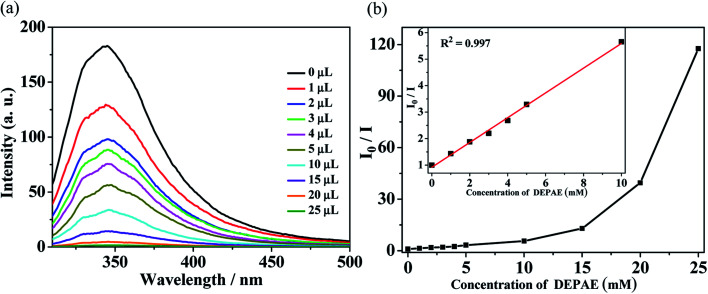
(a) Luminescence spectra and (b) SV curve for Zn-1 by gradual addition of different volume (μL) of pure diethyl phthalate ester (DEPAE) in DMF solution.

**Fig. 5:**
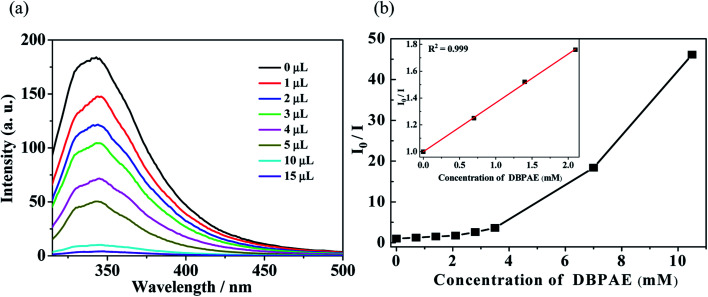
(a) Luminescence spectra and (b) SV curve for Zn-1 by gradual addition of different volume (μL) of pure di-*n*-butyl phthalate ester (DBPAE) in DMF solution.

**Fig. 6:**
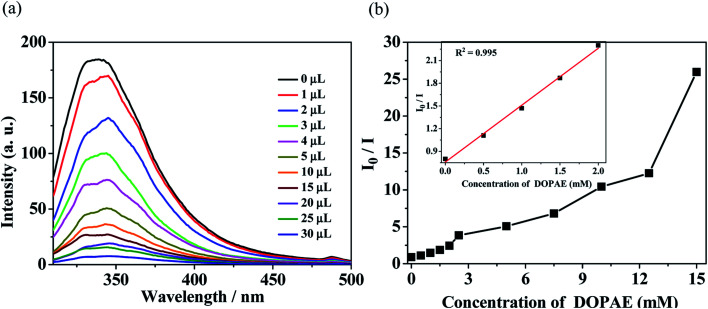
(a) Luminescence spectra and (b) SV curve for Zn-1 by gradual addition of different volume (μL) of pure di-*n*-octyl phthalate ester (DOPAE) in DMF solution.

For the purpose of further investigating the sensing abilities of polymer Zn-1 to PAEs series, the selective sensing tests were conducted by using different amount of PAEs in DMF solution (Fig. 4–6). Suspensions of Zn-1 in DMF solution were prepared by the same method as above mentioned except by gradually increasing the amount of phthalate esters. It is obvious that the fluorescence intensity of DEPAE@Zn-1 weakened dramatically with the increase of diethyl phthalate ester (DEPAE) content and became a half at a low diethyl phthalate ester content of 0.04 vol% (2 mM), and almost completely disappeared at a concentration of 0.4 vol% (20 mM). The quenching percentage of Zn-1 toward diethyl phthalate ester was evaluated to be 99.1%, thus MOF Zn-1 can be seen as a good candidate for highly selectively sensing the DEPAE.

The Stern–Volmer plots of relative luminescent intensity (*I*_0_/*I*) *versus* the concentration of DEPAE were used to quantify the quenching efficiency, which are exhibited in Fig. 4b. The quenching efficiency can be fitted through the equation: *I*_0_/*I* = 1 + *K*_SV_[DEPAE]. *I*_0_ and *I* are the luminescence intensity of Zn-1 before and after addition of the analytes DEPAE, [DEPAE] denotes the molar concentration of DEPAE, and *K*_SV_ is the quenching rate constant. As shown in Fig. 4b, *I*_0_/*I versus* DEPAE concentration plots were almost linear under the condition of low concentration, the quenching constant *K*_SV_ can be determined as 4.67 × 10^2^ M^−1^ (insets of Fig. 4b). Consequently, it can be concluded that DEPAE can be effectively recognized by Zn-1 in a very low concentration range.

In order to probe the fluorescent quenching characteristics of other common PAE series such as DBPAE, DOPAE, a series of experiments were conducted as follows: the samples of Zn-1 (2 mg) was ground and immersed in 5 mL DMF solution and ultrasonicated 20 min to form stable suspensions, and then DBPAE and DOPAE were separately and gradually added to the standard suspensions. The curve of intensity *versus* the concentration of DBPAE and DOPAE are shown in Fig. 5 and 6, respectively. For DBPAE, when the concentration was 2.1 mM, the fluorescence intensity has almost halved, with the quenching efficiency of 43%. At the concentration of 10.5 mM, the luminescence quenching efficiency was as high as 98% (Fig. 5). DOPAE displayed similar property with a quenching efficiency of 47% (at 1.87 mM) and 96% (at 26 mM) (Fig. 6).

What's more, the quenching mechanism of coordination polymer to PAEs was further studied, the UV-absorption spectra of Zn-1 and PAEs in ethanol solution were determined in the range of 200–450 nm. As depicted in Fig. S7,† the excitation peak of Zn-1 overlapped with the absorption band of PAE's, which indicated the competition of absorption of the light source energy between ligand H_2_TIA and PAEs. The energy absorbed by ligand H_2_TIA was transferred to PAEs, which resulted in emission quenching of Zn-1 by means of an excitation energy transfer mechanism.

**Fig. 7:**
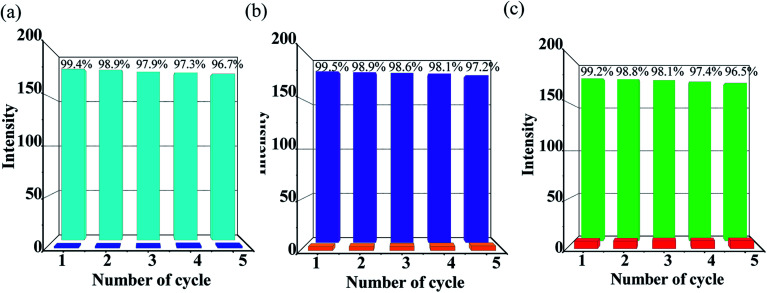
Cyclic response of luminescence intensities at 345 nm of Zn-MOF (Zn-1) for detecting of (a) DEPAE, (b) DBPAE and (c) DOPAE, intensity percentage = *I*/*I*_0_ × 100%.

To measure the quality of a chemosensor, in addition to taking into account the stability, selectivity and sensitivity, the reusability should also be considered as an important factor. Therefore, the tests for the recyclability of Zn-1 to PAEs (DEPAE, DBPAE, DOPAE) were carried out (Fig. 7). The luminescence intensities of Zn-1 could almost restore to their initial level after @PAE-Zn-1 being washed with DMF solvent for a few times. In addition, powder X-ray diffraction experiments further confirmed that the framework of MOF Zn-1 remained unchanged after fluorescence recycling experiments (Fig. S3†). Special stability of the framework and recyclability of MOF Zn-1 indicates the potential applicability as a fluorescent sensor for PAEs organic pollutants.

According to the reported results,^8*c*,9*e*,25^ these multifunctional ligands which contain long π–π conjugated systems have a wide application prospect in fluorescent probing field. Such as the 2D layer complexes {[Cd_2_L_2_(H_2_O)_4_]·H_2_O}_*n*_ and {[Zn_2_L_2_(H_2_O)_4_]·H_2_O}_*n*_ and 3D polymer [Cd_3_L_3_(DMF)_2_]_*n*_ all exhibited highly selective and sensitive chemosensors for Cr^VI^-anions in aqueous medium. In our previous work, we found MOF [Cd_0.5_(TBC)]_*n*_ as a bifunctional luminescence sensor for benzaldehyde and Fe^2+^ ion. In the near future, we will continue to explore the application of these multifunctional ligands in fluorescent probing.

## Conclusion

In conclusion, one porous Zn-MOF (Zn-1) was successfully synthesized by using a multifunctional organic linker H_2_TIA as the building block. Zn-1 exists as a 3,6-connected *rtl* 3D framework with 1D channels, which was composed by binuclear Zn_2_ paddle wheels. In addition, the well-defined correlation between the luminescence intensity of Zn-1 and the concentration of phthalate esters provides a reliable relationship that makes MOF Zn-1 as a low-cost and user-friendly luminescence sensor to determine the concentration of different target species. As far as I know, MOF Zn-1 is the first specialized and efficient luminescence sensor for phthalate esters (carcinogenic organic pollutants). The present study highlights the practical applications of luminescent MOFs as sensors and provides a novel Zn-MOF-based sensor for persistent organic pollutants, which is of great importance for living environments and human health.

## Conflicts of interest

There are no conflicts to declare.

## Supplementary Material

RA-009-C9RA08417G-s001

RA-009-C9RA08417G-s002
