# Supplementary material for: A Zn(ii) metal–organic framework based on bimetallic paddle wheels as a luminescence indicator for carcinogenic organic pollutants: phthalate esters
Source: RSC Adv. 2019 Nov 13;9(63):37101–8. doi: 10.1039/c9ra08417g (PMC9075595; doi:10.1039/c9ra08417g)
Supplement: RA-009-C9RA08417G-s001 [file RA-009-C9RA08417G-s001.pdf]

Electronic Supplementary Material (ESI) for RSC Advances.
This journal is © The Royal Society of Chemistry 2019

Supporting Information

A Zn(II) metal–organic framework based on bimetallic paddle wheels as a luminescence indicator for carcinogenic organic pollutants: phthalate esters

Ming Ze Wu,[†,‡] Zhi Long Ma,[†,‡] Jian Yun Shi,[†,‡] and Li Tian[*,†,‡]

[†] *Tianjin Key Laboratory of Structure and Performance for Functional Molecules.*
[‡] *Key Laboratory of Inorganic-Organic Hybrid Functional Materials Chemistry, Ministry of Education, Colledge of Chemistry, Tianjin Normal University, Tianjin 300387, P. R. China. E-mail: lilytianli@hotmail.com*

Table of Contents

Section S1	Crystallographic Data	2
Section S2	Infrared Spectrum (IR)	3
Section S3	Powder X-ray Diffraction (PXRD)	3–4
Section S4	Thermal Gravimetric Analysis Curves	4
Section S5	Sorption Properties	5
Section S6	Other Luminescence Properties	5
Section S7	UV–vis Spectra	6
Section S8	Tables of Crystal Data	6

1 Crystallographic Data

X-Ray crystallography. Crystallographic data were collected at 296 K (for **Zn-1**) on an Agilent SuperNova (Dual, Cu at zero, AtlasS2, CCD) diffractometer equipped with graphite-monochromated Mo-Kα radiation (λ = 0.71073 Å), using the *φ–ω* scan technique. Semiempirical multiscan absorption corrections were applied by SCALE3 ABSPACK, and the programs CrysAlisPro were used for integration of the diffraction profiles. The structures were solved by direct methods with the ShelXT-2013 structure solution program and refined using least squares minimization by with the ShelXL-2013 refinement package. Some restraints are employed, such as ISOR (anisotropic parameter), DFIX (restricting the distance between two atoms) to solve the disorder of the O atoms. All non-hydrogen atoms were refined anisotropically, and hydrogen atoms were located geometrically and refined isotropically. Crystallographic data are listed in Table S1.

Table S1. Crystallographic Data and Structure Refinement Details for **Zn-1**

	**1**
formula	$C_{14}H_{14}N_4O_5Zn$
Mr	383.66
crystal system	monoclinic
space group	$P2_1/c$
a (Å)	10.852(3)
b (Å)	11.898(3)
c (Å)	15.037(3)
α(°)	90
β(°)	110.289(3)
γ(°)	90
V (Å^3)	1821.2(7)
Z	4
ρcalc (Mg/m^3)	1.399
μ (mm^{-1})	1.377
F(000)	784
θ range(°)	2.264~25.01
limiting indices	$-13 \leq h \leq 13$, $-14 \leq k \leq 11$, $-18 \leq l \leq 18$
Reflns collected	11253
GOF on F^2	1.114
R_1/wR_2 [$I > 2\sigma(I)$]	$R_1 = 0.0890$, $wR_2 = 0.2524$
R_1/wR_2 (all data)	$R_1 = 0.1005$, $wR_2 = 0.2637$

2 Infrared Spectrum (IR)

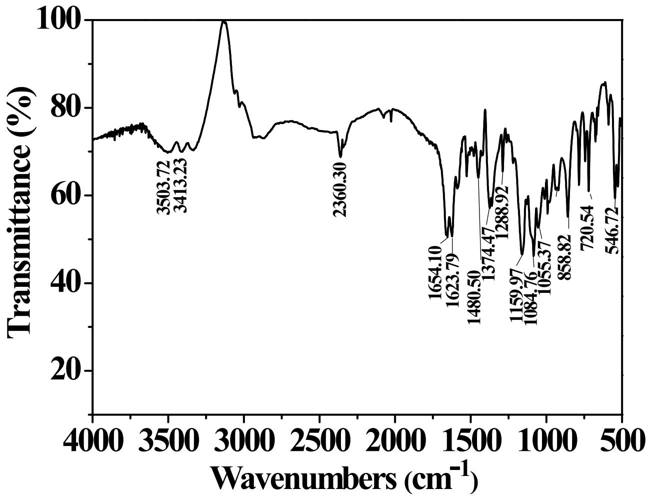

Fig. S1. Infrared Spectrum (IR) of complex **Zn-1**.

3 Powder X-ray Diffraction

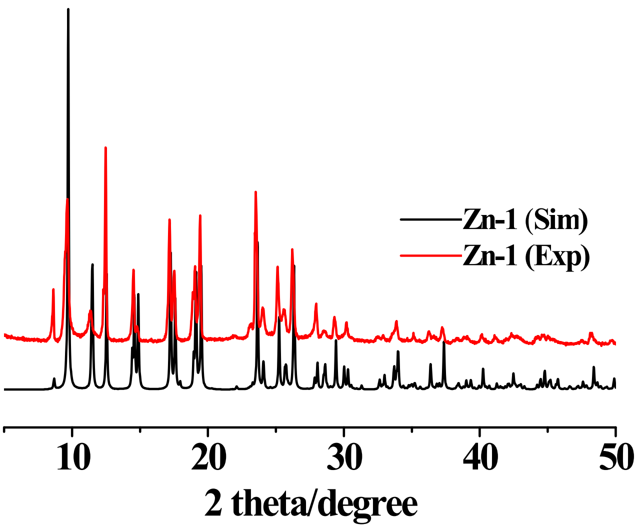

Fig. S2. XRD patterns of complex **Zn-1**.

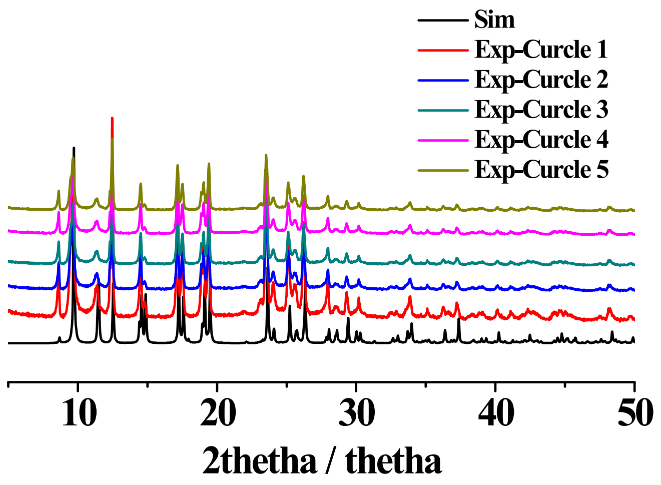

Fig. S3. XRD patterns of **Zn-1** toward DEPAE after cycle experiments.

4 Thermal Gravimetric Analysis Curves

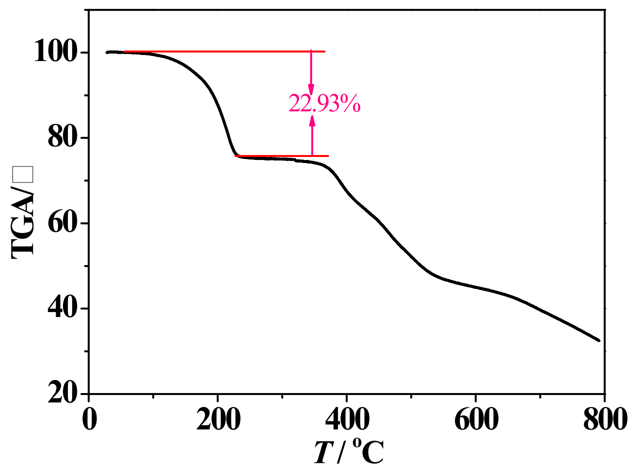

Fig. S4. TGA curves of complex **Zn-1**.

5 Sorption Properties

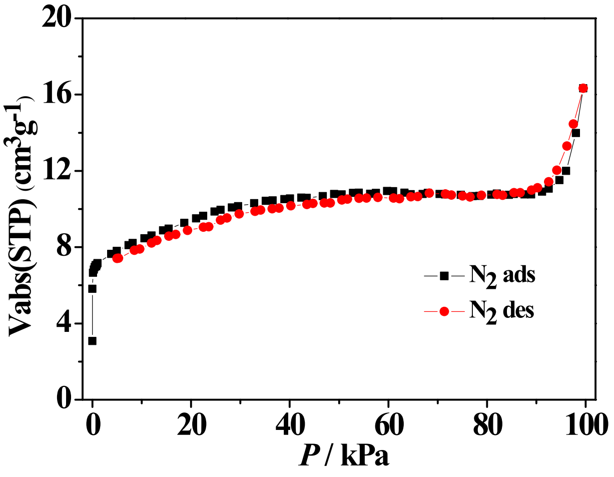

Fig. S5. N_2 sorption isotherms of **Zn-1** at 77 K

6 Other Luminescence Properties

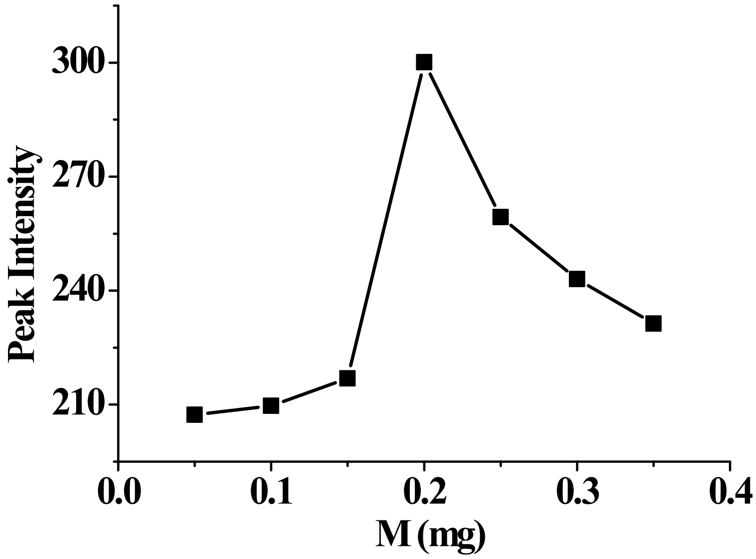

Fig. S6. The luminescence intensities at 345 nm at different amount of coordination polymer **Zn-1** (mg) in 10 ml DMF solution when excited at 290 nm.

7 UV–vis Spectra

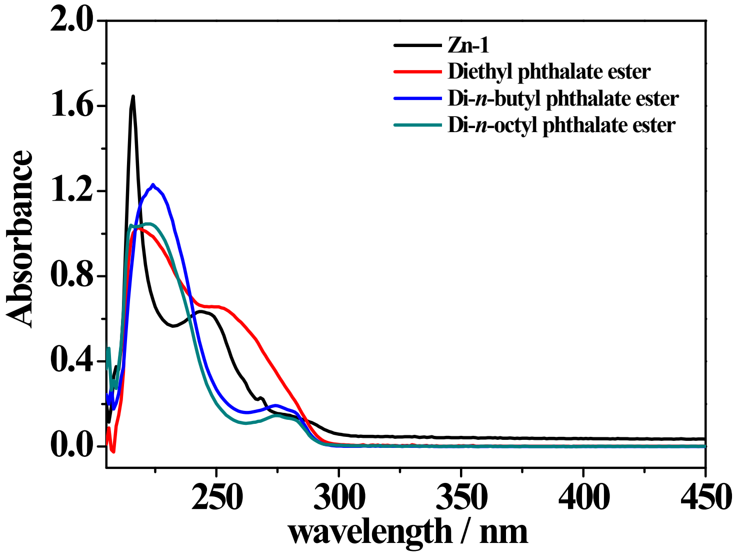

Fig. S7. UV–vis spectra of the three phthalate esters and coordination polymer **Zn-1**.

8 Tables of Crystal Data

Table S2. Selected bond lengths (Å) and bond angles (°) in complex **Zn-1**.

Zn(1)-O(1)#1	2.017(4)	O(4)#2-Zn(1)-N(1)	98.8(2)
Zn(1)-O(4)#2	2.027(4)	O(2)#3-Zn(1)-N(1)	102.6(2)
Zn(1)-O(2)#3	2.032(4)	O(1)#1-Zn(1)-O(3)#4	87.9(2)
Zn(1)-N(1)	2.043(5)	O(4)#2-Zn(1)-O(3)#4	158.33(18)
Zn(1)-O(3)#4	2.044(4)	O(2)#3-Zn(1)-O(3)#4	90.48(19)
O(1)#1-Zn(1)-O(4)#2	85.5(2)	N(1)-Zn(1)-O(3)#4	102.6(2)
O(1)#1-Zn(1)-O(2)#3	158.40(18)	O(1)#1-Zn(1)-Zn(1)#5	75.87(13)
O(4)#2-Zn(1)-O(2)#3	88.2(2)	O(4)#2-Zn(1)-Zn(1)#5	77.78(13)
O(1)#1-Zn(1)-N(1)	98.7(2)	N(1)-Zn(1)-Zn(1)#5	173.72(15)

#1: -*x*+1, -*y*, -*z*; #2: -*x*+1, *y* -1/2, -*z*+1/2; #3: *x*-1, *y*, *z*; #4: *x*-1, -*y*+1/2, *z*-1/2.
